# Effect of radiation therapy on patients with stage T3 or T4 malignant phyllodes tumors: a retrospective observational study based on SEER

**DOI:** 10.1007/s00432-023-05517-0

**Published:** 2023-12-28

**Authors:** Gongyin Zhang, Ping Yang, Jinsheng Zeng, Changlong Wei

**Affiliations:** 1 Department of Breast Surgery, The First Affiliated Hospital of Nanchang University, Nanchang, Jiangxi China; 2 Department of Radiation Oncology, The First Affiliated Hospital of Dalian Medical University, Dalian, Liaoning China

**Keywords:** Malignant phyllodes tumors, Radiotherapy, Overall survival (OS), Breast cancer-specific survival (BCSS)

## Abstract

**Purpose:**

Among all primary breast tumors, malignant phyllodes tumor of the breast (MPTB) make up less than 1%. In the treatment of phyllode tumors, surgical procedures such as mastectomy and breast-conserving surgery are the mainstay. MPTB has, however, been controversial when it comes to treating it with RT. We aimed to explore the prognostic impact of RT and other clinicopathologic factors on long-term survival for patients with stage T3 or T4 malignant phyllodes tumors.

**Methods:**

We select patients with stage T3 or T4 MPTB who qualified for the criteria between 2000 and 2018 via the Surveillance, Epidemiology, and End Results (SEER) database. We performed 1:1 propensity score matching (PSM) and Kaplan–Meier analysis to explore the role of RT in long-term survival of patients with stage T3 or T4 MPTB. A univariate and multivariate analysis of breast cancer-specific survival (BCSS) and overall survival (OS) risk factors was carried out using a Cox proportional hazards model. In addition, the nomogram graph of OS and BCSS was constructed.

**Results:**

A total of 583 patients with stage T3 or T4 malignant phyllodes tumors were included in this study, of whom 154 (26.4%) received RT, and 429 (73.6%) were treated without RT. Before adjustment, between groups with and without RT, BCSS (*p* = 0.1) and OS (*p* = 0.212) indicated no significant difference respectively. Using of PSM, the two groups still did not differ significantly in BCSS (*p* = 0.552) and OS (*p* = 0.172). In multivariate analysis, age (*p* < 0.001), surgery of primary site (*p* < 0.001) and distant metastatic status (*p* < 0.001) were related to prognosis, while RT still did not affect BCSS (*p* = 0.877) and OS (*p* = 0.554).

**Conclusion:**

Based on the SEER database analysis, the study suggests that the patients with stage T3 or T4 MPTB treated with RT after surgery didn't have significant differences in BCSS or OS compared to those not treated with RT.

## Introduction

Phyllodes tumor of the breast (PTB) are rare fibroepithelial neoplasms, occupies less than 1% of all primary breast tumors. They are most commonly found in women aged between 45 and 50 year (Mishra et al. 2013) and often unilateral. Based on the number of mesenchymal cells, cell heterogeneity, nuclear division, tumor margins, and the presence or absence of stroma in the tumor, the World Health Organization (WHO) divided them into three levels: benign, borderline, and malignant. Of these, malignant phyllodes tumor of the breast (MPTB) account for about one-fourth of PTBs (Zhang and Kleer 2016) and about 0.5% of all malignant breast tumors. MPTB manifests as an insidious disease that progresses rapidly. Typically, the lesions are unilateral, solitary, nodular, and painless tumor between one centimeter and 40 cm in diameter (Hawkins et al. 1992). A high rate of local recurrence and distant metastasis is characteristic of MPTB (Kapiris et al. 2001). It has been reported that benign, borderline and malignant phyllodes tumors recur at rates of 10–17%, 14–25%, 23–30% (Lu et al. 2019), respectively. For the distant metastasis, the predominant mode of metastasis of MPTB is hematogenous and rarely lymph node metastasis. Lung, bone, and abdominal viscera are the most common sites of distant disease (Parker and Harries 2001). The surgical approach is the mainstay of treatment for PTB. However, radiation therapy (RT) remains controversial in the treatment of phyllodes tumors (Chaney et al. 2000; Macdonald et al. 2006; Belkacemi et al. 2008; Pezner et al. 2008; Barth et al. 2009). In a previous study of MPTB patients, RT led to poorer treatment outcomes (Macdonald et al. 2006). In an article by Zhao et al., it was shown that RT resulted in a prolonged disease-specific survival time and overall survival time for patients with malignant tumors (Zhou et al. 2018). Therefore, despite the increasing use of RT in malignant phyllodes tumors, the role of RT is still unclear.

Therefore, based on the Surveillance, Epidemiology, and End Results (SEER) database, the aim of this study was to evaluate the impact of RT and other clinicopathologic factors on breast cancer-specific survival (BCSS) and overall survival (OS) for patients with stage T3 or T4 MPTB to explore the value of RT in patients with MPTB.

## Methods

### Study population

In the SEER database, we conducted a retrospective study of MPTB patients between 2000 and 2018. Patients with MPTB were required to meet the following inclusion criteria: women with stage T3 or T4 malignant phyllodes tumors (ICD-O-39020/3), diagnosed between 2000 and 2018. We excluded patients with incomplete follow-up information, such as those without information of RT. The age at diagnosis, race, marital status, laterality, T stage (T3-4), lymph node status, distant metastatic status, tumor grade, long-term survival, death status, surgery of primary site, local lymphatic biopsy, chemotherapy (CT) and RT were extracted from the SEER database. The corresponding author can provide the data.

### Statistical analysis

In our study, we used Chi-square tests to compare the baseline characteristics of patients who underwent RT and those without. In order to control for selection bias, we performed one-to-one propensity score matching (PSM). The primary endpoint measures evaluated were BCSS and OS. The BCSS was regarded as the period of time between tumor diagnosis and death due to MPTB. The OS was regarded as the time between tumor diagnosis and any reason of death. Based on Kaplan–Meier analysis and log rank comparison, we compared OS and BCSS between the two groups. A univariate and multivariate analysis of OS and BCSS risk factors was carried out using a Cox proportional hazards model. In addition, the nomogram graph of OS and BCSS was constructed. The analyses were conducted using R language software. A *p* value < 0.05 was assumed to be statistically significant.

## Results

### Characteristics

A total of 583 patients with stage T3 or T4 malignant phyllodes tumors were included in this study, of whom 154 (26.4%) received RT, and 429 (73.6%) were treated without RT. As shown in Table 1, patients who received RT were more likely to receive CT (*p* = 0.007), biopsy of lymph nodes (*p* = 0.043), and be in G3 or G4 grades (*p* = 0.003), compared to those who did not receive RT. As a result of PSM balancing the differences in characteristics, 154 pairs of patients were analyzed (Table 1). The difference between the two groups was not significant.

**Table 1:** Baseline characteristics

	Unadjusted analysis			Propensity score matching		
	Non-RT = 429	RT = 154	*p* value	Non-RT = 154	RT = 154	*p* value
Age			1.000			0.731
< 50	197 (45.9%)	71 (46.1%)		67 (43.5%)	71 (46.1%)	
≥ 50	232 (54.1%)	83 (53.9%)		87 (56.5%)	83 (53.9%)	
Race			0.311			0.803
White	298 (69.5%)	98 (63.6%)		97 (63.0%)	98 (63.6%)	
Black	51 (11.9%)	25 (16.2%)		22 (14.3%)	25 (16.2%)	
Others	80 (18.6%)	31 (20.1%)		35 (22.7%)	31 (20.1%)	
Marital status			0.203			1.000
Married	198 (46.2%)	72 (46.8%)		73 (47.4%)	72 (46.8%)	
Unmarried	200 (46.6%)	77 (50.0%)		77 (50.0%)	77 (50.0%)	
Unknown	31 (7.23%)	5 (3.25%)		4 (2.60%)	5 (3.25%)	
Tumor grade			0.003			0.984
G1	39 (9.09%)	10 (6.49%)		11 (7.14%)	10 (6.49%)	
G2	40 (9.32%)	6 (3.90%)		6 (3.90%)	6 (3.90%)	
G3	54 (12.6%)	35 (22.7%)		38 (24.7%)	35 (22.7%)	
G4	43 (10.0%)	23 (14.9%)		20 (13.0%)	23 (14.9%)	
Unknown	253 (59.0%)	80 (51.9%)		79 (51.3%)	80 (51.9%)	
Laterality			0.355			1.000
Left	194 (45.2%)	77 (50.0%)		76 (49.4%)	77 (50.0%)	
Right	235 (54.8%)	77 (50.0%)		78 (50.6%)	77 (50.0%)	
Tumor stage			1.000			1.000
T3	383 (89.3%)	137 (89.0%)		137 (89.0%)	137 (89.0%)	
T4	46 (10.7%)	17 (11.0%)		17 (11.0%)	17 (11.0%)	
Lymph node			0.116			1.000
Negative	422 (98.4%)	148 (96.1%)		149 (96.8%)	148 (96.1%)	
Positive	7 (1.63%)	6 (3.90%)		5 (3.25%)	6 (3.90%)	
Distant metastasis			0.189			0.571
Negative	418 (97.4%)	146 (94.8%)		149 (96.8%)	146 (94.8%)	
Positive	11 (2.56%)	8 (5.19%)		5 (3.25%)	8 (5.19%)	
Surgery of primary site			0.387			0.682
No surgery	4 (0.93%)	1 (0.65%)		1 (0.65%)	1 (0.65%)	
BCS	150 (35.0%)	45 (29.2%)		37 (24.0%)	45 (29.2%)	
Mastectomy	275 (64.1%)	108 (70.1%)		116 (75.3%)	108 (70.1%)	
Chemotherapy			0.007			0.524
No	415 (96.7%)	140 (90.9%)		144 (93.5%)	140 (90.9%)	
Yes	14 (3.26%)	14 (9.09%)		10 (6.49%)	14 (9.09%)	
Local lymphatic biopsy			0.043			1.000
No	294 (68.5%)	91 (59.1%)		90 (58.4%)	91 (59.1%)	
Yes	135 (31.5%)	63 (40.9%)		64 (41.6%)	63 (40.9%)	

### Survival analysis of radiotherapy

**Fig. 1:**
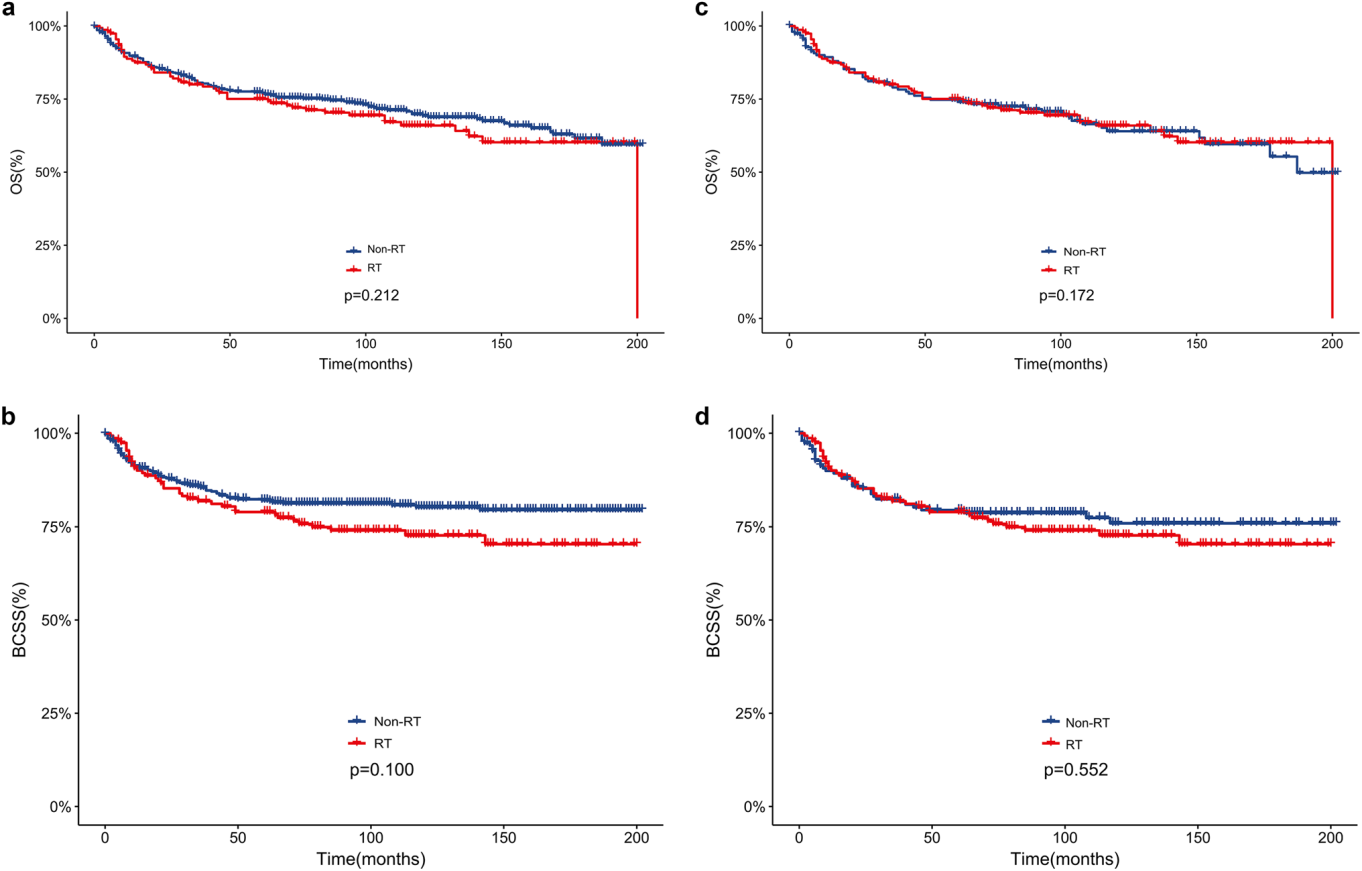
**a**–**d** Comparisons of OS and BCSS between RT and Non-RT before and after PSM

Before adjustment (Fig. 1a, b), neither BCSS (*p* = 0.1) nor OS (*p* = 0.212) differed significantly between groups with and without RT. Using of PSM (Fig. 1c, d), the two groups still did not differ significantly in BCSS (*p* = 0.552) and OS (*p* = 0.172). Similarly, among the multivariate Cox regression analysis, RT still had no effect on BCSS (*p* = 0.877) and OS (*p* = 0.554). Apparently, in this study, the patients with stage T3 or T4 MPTB did not benefit from RT in terms of OS and BCSS.

### COX regression analysis

An univariate Cox regression analysis (Table 2) showed the following results for BCSS: higher grade of tumor differentiation (*p* = 0.009, HR = 3.32, 95% CI = 1.35–8.19), T stage (*p* < 0.001, HR = 3.38, 95% CI = 2.21–5.18), lymph node status (*p* = 0.005, HR = 3.23, 95% CI = 1.42–7.35) distant metastasis (*p* < 0.001, HR = 0.03, 95% CI = 0.02–0.06), local lymphatic biopsy (*p* = 0.045, HR = 1.46, 95% CI = 1.01–2.10), surgery of primary site (*p* < 0.001, HR = 0.03, 95% CI = 0.01–0.08 in BCS and *p* < 0.001, HR = 0.08, 95%CI = 0.03–0.20 in mastectomy), CT (*p* < 0.001, HR = 4.45, 95% CI = 2.58–7.66) were related to BCSS. In addition, multivariate Cox regression analysis (Table 2) indicated that distant metastasis (*p* < 0.001, HR = 0.071, 95% CI = 0.034–0.147), surgery of primary site (*p* < 0.001, HR = 0.059, 95% CI = 0.018–0.200 in BCS and *p* < 0.001, HR = 0.119, 95%CI = 0.038–0.368 in mastectomy) were independent risk factors for BCSS.

**Table 2:** Univariate and multivariate analysis of risk factors for BCSS

	Univariate analysis		Multivariate analysis	
	HR (95%CI)	*P*	HR (95%CI)	*P*
Age				
< 50	1		1	
≥ 50	1.29 (0.89, 1.86)	0.177	1.30 (0.878, 1.93)	0.189
Race				
White	1		1	
Black	1.26 (0.76, 2.08)	0.368	1.12 (0.68, 2.05)	0.553
Other	1.07 (0.67, 1.71)	0.761	0.88 (0.52, 1.48)	0.635
Marital status				
Married	1		1	
Unmarried	1.08 (0.75, 1.57)	0.669	0.913 (0.614, 1.359)	0.655
Unknown	0.86 (0.37, 1.99)	0.721	0.915 (0.386, 2.167)	0.840
Tumor grade				
G1	1		1	
G2	1.07 (0.34, 3.30)	0.913	1.06 (0.34, 3.32)	0.920
G3	2.43 (0.99, 5.97)	0.053	1.41 (0.56, 3.55)	0.470
G4	3.32 (1.35, 8.19)	0.009	2.21 (0.87, 5.59)	0.095
Unknown	1.66 (0.72, 3.84)	0.237	1.25 (0.53, 2.93)	0.611
Laterality				
Left	1		1	
Right	0.86 (0.60, 1.24)	0.429	0.91 (0.62, 1.34)	0.644
Tumor stage				
T3	1		1	
T4	3.38 (2.21, 5.18)	< 0.001	1.58 (0.96, 2.60)	0.070
Lymph node				
Negative	1		1	
Positive	3.23 (1.42, 7.35)	0.005	0.970 (0.36, 2.60)	0.951
Distant metastasis				
Negative	1		1	
Positive	0.03 (0.02, 0.06)	< 0.001	0.071 (0.034, 0.147)	< 0.001
Surgery of primary site				
No surgery	1		1	
BCS	0.03 (0.01, 0.08)	< 0.001	0.059 (0.018, 0.2)	< 0.001
Mastectomy	0.08 (0.03, 0.20)	< 0.001	0.119 (0.038, 0.368)	< 0.001
Radiotherapy				
No	1		1	
Yes	1.38 (0.94, 2.03)	0.100	1.03 (0.68, 1.56)	0.877
Chemotherapy				
No	1		1	
Yes	4.45 (2.58, 7.66)	< 0.001	1.419 (0.721, 2.794)	0.311
Local lymphatic biopsy				
No	1		1	
Yes	1.46 (1.01, 2.10)	0.045	1.117 (0.753, 1.656)	0.582

An univariate Cox regression analysis (Table 3) displayed the following results for OS that age (*p* < 0.001, HR = 1.82, 95% CI = 1.33–2.48), T stage (*p* < 0.001, HR = 2.97, 95% CI = 2.06–4.29), distant metastasis (*p* < 0.001, HR = 0.03, 95% CI = 0.02–0.06), lymph node status (*p* = 0.003, HR = 2.93, 95% CI = 1.44–5.98), local lymphatic biopsy (*p* = 0.002, HR = 1.59, 95% CI = 1.18–2.14), surgery of primary site (*p* < 0.001, HR = 0.04, 95% CI = 0.01–0.09 in breast-conserving surgery (BCS) and *p* < 0.001, HR = 0.10, 95% CI = 0.04–0.24 in mastectomy) and CT (*p* < 0.001, HR = 4.44, 95% CI = 2.81–7.02) were related to OS. Additionally, the multivariate Cox regression analysis (Table 3) revealed that age (*p* < 0.001, HR = 1.799, 95% CI = 1.292–2.504), distant metastasis (*p* < 0.001, HR = 0.079, 95% CI = 0.041–0.051), surgery of primary site (*p* < 0.001, HR = 0.051, 95% CI = 0.017–0.154 in BCS and *p* < 0.001, HR = 0.106, 95% CI = 0.037–0.302 in mastectomy) were independent risk factors for OS.

**Table 3:** Univariate and multivariate analysis of risk factors for OS

	Univariate analysis		Multivariate analysis	
	HR (95%CI)	p	HR (95%CI)	p
Age				
< 50	1		1	
≥ 50	1.82 (1.33, 2.48)	< 0.001	1.799 (1.292, 2.504)	< 0.001
Race				
White	1		1	
Black	1.17 (0.77, 1.78)	0.454	1.202 (0.767, 1.884)	0.423
Others	1.03 (0.71, 1.51)	0.868	0.895 (0.59, 1.36)	0.602
Marital status				
Married	1		1	
Unmarried	1.24 (0.91, 1.68)	0.172	1.082 (0.782, 1.496)	0.633
Unknown	1.23 (0.67, 2.26)	0.509	1.182 (0.633, 2.210)	0.599
Tumor grade				
G1	1		1	
G2	0.91 (0.42, 2.00)	0.817	0.903 (0.408, 1.996)	0.800
G3	1.73 (0.91, 3.27)	0.092	1.09311 (0.56402, 2.1186)	0.792
G4	1.82 (0.94, 3.53)	0.076	1.24171 (0.62583, 2.4637)	0.536
Unknown	1.18 (0.66, 2.11)	0.575	0.944 (0.522, 1.709)	0.850
Laterality				
Left	1		1	
Right	0.89 (0.66, 1.20)	0.443	0.969 (0.709, 1.323)	0.842
Tumor stage				
T3	1		1	
T4	2.97 (2.06, 4.29)	< 0.001	1.445 (0.953, 2.189)	0.083
Lymph node				
Negative	1		1	
Positive	2.93 (1.44, 5.98)	0.003	1.238 (0.547, 2.803)	0.6084
Distant metastasis				
Negative	1		1	
Positive	0.03 (0.02, 0.06)	< 0.001	0.079 (0.041, 0.151)	< 0.001
Surgery of primary site				
No surgery	1		1	
BCS	0.04 (0.01, 0.09)	< 0.001	0.051 (0.017, 0.154)	< 0.001
Mastectomy	0.10 (0.04, 0.24)	< 0.001	0.106 (0.037, 0.302)	< 0.001
Radiotherapy				
No	1		1	
Yes	1.16 (0.84, 1.61)	0.373	0.899 (0.634, 1.276)	0.554
Chemotherapy				
No	1		1	
Yes	4.44 (2.81, 7.02)	< 0.001	1.921 (1.090, 3.387)	0.424
Local lymphatic biopsy				
No	1		1	
Yes	1.59 (1.18, 2.14)	0.002	1.227 (0.893, 1.686)	0.208

### Construction of the prognostic model for OS and BCSS

**Fig. 2:**
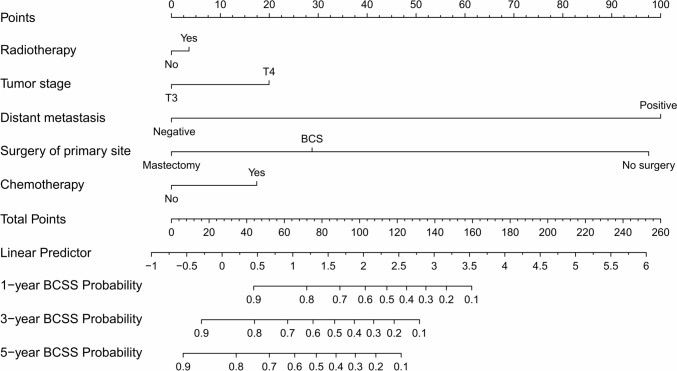
Radiotherapy, T stage, distant metastases, surgery of primary site, and chemotherapy are included in the nomogram model for predicting 1-, 3-, and 5-years BCSS rates of patients with stage T3-4 MPTB

**Fig. 3:**
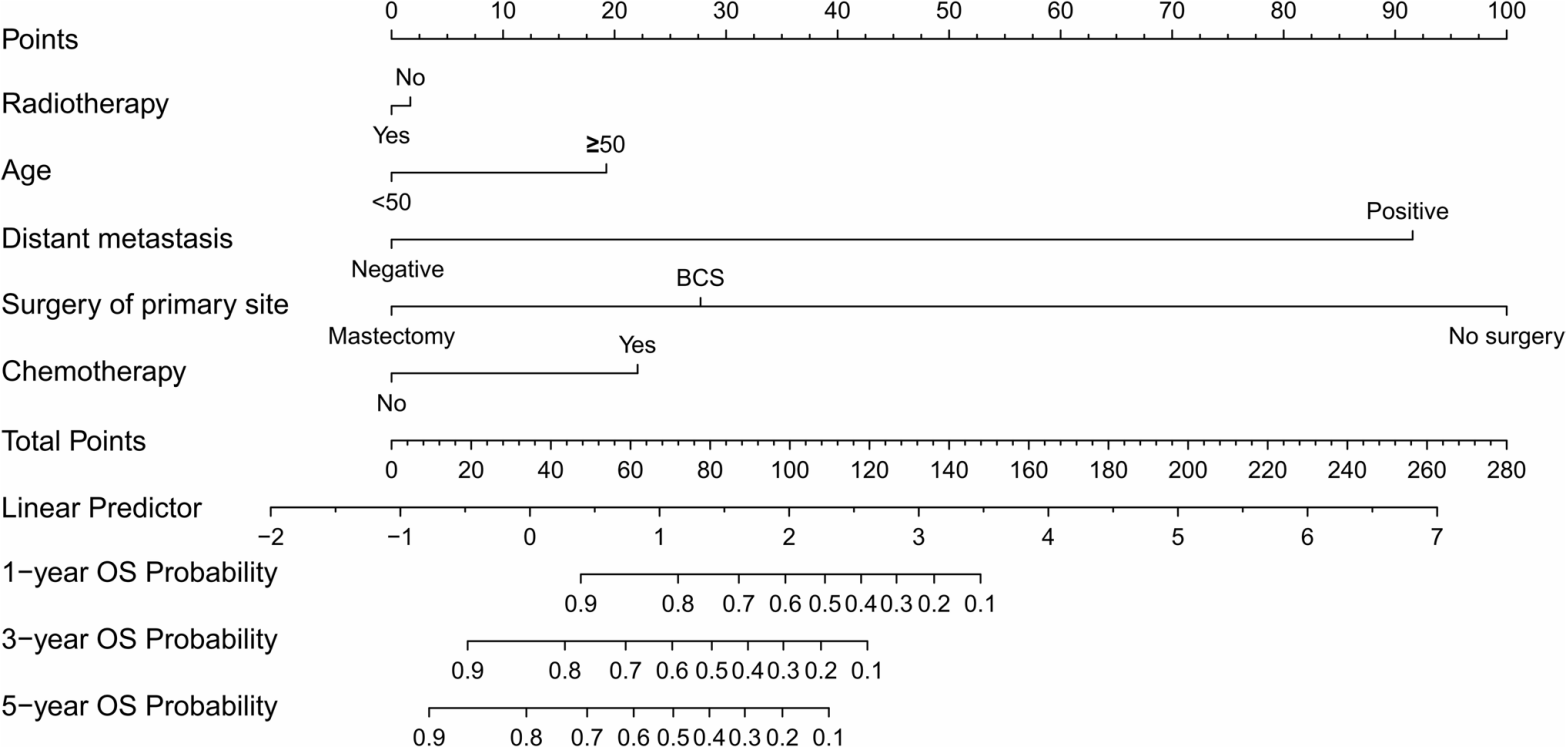
Radiotherapy, age, distant metastases, surgery of primary site, and chemotherapy are included in the nomogram model for predicting 1-, 3-, and 5-year OS rates of patients with stage T3-4 MPTB

With the aim of predicting the 1-, 3-, and 5-years OS and BCSS for patients with stage T3 or T4 MPTB, we combined age, T stage, distant metastases, surgical removal of the primary site, radiotherapy, and chemotherapy. Prognostication factors for BCSS (Fig. 2) and OS (Fig. 3) can be scored on a score scale in the model. Patients with stage T3 or T4 MPTB can be predicted to have an overall survival of 1-, 3-, and 5-years based on the total of these scores. In one case, a middle-aged woman with MPTB and a stage T4 tumor had a BCSS and OS score of 70 and 60, respectively. Consequently, OS rates were assessed to be 85%, 68%, and 57% for patient’s 1-, 3-, and 5-years individually. Similarly, BCSS rates were assessed to be 82%, 65%, and 54% for patient’s 1-, 3-, and 5-years individually.

## Discussion

Among all primary breast tumors, MPTB make up less than 1%. In clinical practice, phyllodes tumors are characterized by a single, mobile, round tumor with painless, progressive enlargement, and are often the main reason for patients visit. According to statistics, the number of patients with MPTB more than doubled from 830 to 1980 in the 11 years from 2002 to 2013 (Macdonald et al. 2006). It seems that patients with MPTB are growing in number. The mainstays of curative treatment of phyllodes tumors are surgical procedures, such as breast-conserving surgery (BCS) and mastectomy. In spite of this, MPTB are associated with a high risk of local recurrence. In a study of 5,530 patients with phyllodes tumors, the overall recurrence rate was 19.4%, while malignant phyllodes tumors recurred at a rate of 26% (Spitaleri et al. 2013). As well, MPTB is still related to high mortality and metastasis rates (Kapiris et al. 2001; Asoglu et al. 2004; Fou et al. 2006; Onkendi et al. 2014). Thus, the local control and prevention of metastasis after surgery for MPTB is particularly important.

There has been an increase in the use of RT in recent years. Commission on Cancer of the American College of Surgeons’ National Cancer Databases show that 19.5% of patients receiving RT for phyllodes tumors in 2008–2009 received RT, a substantial increase over the 9.5% rate in 1998–1999 (Gnerlich et al. 2014). Phyllodes tumors, however, remain controversial when it comes to RT. Previous studies have demonstrated the ability of RT to reduce local recurrence after surgery for MPTB (Belkacemi et al. 2008; Barth et al. 2009). Nevertheless, the decrease in local recurrence is not associated with a significant improvement in long-term survival after RT (Neron et al. 2020). In this study, both before and after adjustment, according to our survival analyses, RT did not affect long-term survival for patients with stage T3 or T4 malignant phyllodes tumors (Fig. 1). Before adjustment, no significant difference in OS (*p* = 0.212) and BCSS (*p* = 0.1) was found between groups with and without RT. Using of PSM, there was still no significant difference between the two groups in OS (*p* = 0.172) or BCSS (*p* = 0.552). It is worth noting that the survival curve (Fig. 1) shows that the rate of long-term survival in the non-RT group is higher than that in the RT group, although there is no difference between the two groups. Univariate and multivariate Cox regression analyses in this study also showed (Table 2 and Table 3) that RT did not affect BCSS (*p* = 0.1, HR = 1.38, 95% CI = 0.94–2.03 in the univariate Cox regression analyses and *p* = 0.877, HR = 1.03, 95% CI = 0.68–1.56 in the multivariate Cox regression analyses) and OS (*p* = 0.373, HR = 1.16, 95% CI = 0.84–1.61 in the univariate Cox regression analyses and *p* = 0.554, HR = 0.899, 95% CI = 0.634–1.276 in the multivariate Cox regression analyses). In the previous literature, similar results have been confirmed. According to Pandey et al., the addition of RT did not improve 5-year disease-free survival (Pandey et al. 2001). It was concluded by Confavreux et al. that the effects of RT on long-term survival in MPTB patients were insignificant, and even unneeded RT may even have been harmful in some cases (Confavreux et al. 2006). Of course, there is evidence in the literature that MPTB patients with these types such as those who are younger (< 45 years), those with larger tumors, and those with more extensive resections are more likely to benefit from RT (Chao et al. 2019). At least, the outcome of this study of patients with stage T3 or T4 MPTB revealed no significant effect of RT on BCSS and OS, which is consistent with previous findings.

In our study, an analysis of univariate and multivariate data showed that patients with stage T3 or T4 MPTB had poor survival rates when their age ≥ 50 years. Age ≥ 50 years in MPTB may be associated with the risk of metastasis (Neron et al. 2020). Regarding the prognostic impact of surgical approach, the survival rates from BCS and mastectomy were similar in one study (Mitus et al. 2014). Nevertheless, in a study of patients with stage T1-2 MPTB, BCS was associated with better OS and BCS (Chen and Ya 2023). This may be due to the fact that BCS can lead to better cosmetic results, higher quality of life, and other benefits. In our subgroup of patients with stage T3 or T4 MPTB, the surgery of primary site significantly affected OS and BCSS from univariate and multivariate Cox regression analyses. As the nomogram model (Figs. 2 and 3) shows, mastectomy is more favorable for patients’ long-term survival. Since patients with stage T3 or T4 MPTB may have more unfavorable features, mastectomy is the preferred surgical approach. Moreover, few studies have shown that long-term survival for MPTB patients can be enhanced by CT. CT was found to have little effect on survival of patients in a prospective study (Ramakant et al. 2015). It is important to note, however, that the sample size of this literature is small. Furthermore, an earlier study by Broglio K et al. concluded CT had no effect on survival (Morales-Vasquez et al. 2007). In our study, multivariate Cox regression analyses showed no significant effect of CT (*p* = 0.311, HR = 1.419, 95% CI = 0.721–2.794 in the BCSS and *p* = 0.424, HR = 1.921, 95% CI = 1.090–3.387 in OS) on long-term survival in patients with stage T3 or T4 MPTB, which is in accordance with previous studies. It is recommended that CT can be considered in extreme cases, such as when the tumor is large or invades structures such as the chest wall (Strode et al. 2017). In conclusion, chemotherapy is not recommended as first-line treatment for phyllodes tumors so far.

In this study, there are several limitations. Firstly, in the SEER database, borderline phyllodes tumors may be incorrectly coded as malignant diseases, affecting the results of the analysis. Secondly, approximately half of the patients had no tumor grade reported. Thirdly, it is also necessary for the SEER database to be continuously improved, as it does not provide data on local recurrence and histopathological, including resection margin status. Therefore, we could not explore the impact of RT in recurrence of MPTB.

## Conclusions

Based on the SEER database analysis, the study suggests that the patients with stage T3 or T4 MPTB treated with RT after surgery didn't have significant differences in BCSS or OS compared to those not treated with RT. However, the value of RT in MPTB still needs to be validated based on adequate data from large prospective studies.

## Data Availability

The data supporting the findings of this study can be found at http://seer.cancer.gov, a database maintained by the Surveillance, Epidemiology, and End Results (SEER) program.
